# Moderate Dose Bovine Colostrum Supplementation in Prevention of Upper Respiratory Tract Infections in Medical University Students: A Randomized, Triple Blind, Placebo-Controlled Trial

**DOI:** 10.3390/nu15081925

**Published:** 2023-04-16

**Authors:** Magdalena Baśkiewicz-Hałasa, Ewa Stachowska, Elżbieta Grochans, Dominika Maciejewska-Markiewicz, Leonard Bühner, Karolina Skonieczna-Żydecka, Maciej Hałasa

**Affiliations:** 1 Department of General Pathology, Pomeranian Medical University in Szczecin, Powstańców Wlkp. 72, 70-111 Szczecin, Polandmaciupam@op.pl (M.H.); 2 Department of Human Nutrition and Metabolomics, Pomeranian Medial University in Szczecin, Broniewskiego 24, 71-460 Szczecin, Poland; 3 Department of Nursing, Pomeranian Medical University in Szczecin, Żołnierska 48, 71-210 Szczecin, Poland; 4 Department of Biochemical Science, Pomeranian Medical University in Szczecin, Broniewskiego 24, 71-460 Szczecin, Poland

**Keywords:** infection, colostrum, trial

## Abstract

Colostrum supplementation has been confirmed to protect from upper respiratory tract infections (URTIs) in athletes. Our trial was designed to find out whether other young adults who have potentially been exposed to increased risk of developing URTIs can also benefit. Homogenous population of medical (MED) students (at risk) and health science (HSci) peers were supplemented with a relatively low dose (0.5–1.0 g/day) of bovine colostrum (COL) or placebo (PBO) over 45 days and then once again over 7 days starting at day 87. The trial lasted 107 days. Subjects were monitored solely by them filling out online daily questionnaires containing questions about frequency and severity of URTIs symptoms, well-being, and potential gastrointestinal side-effects. A significant level of protection from URTIs was observed as expressed by dropping frequency of symptomatic days in COL vs. PBO group among MED vs. HSci students. The same effect was also recorded for severity of symptoms, as well as general well-being perception. Overall, it can be concluded that although young healthy people seem to have sufficient defenses from URTIs, COL supplementation can provide significant support in such protection among those at higher infectious risk because of exposure to a heavy workload and increased contact with infectious agents.

## 1. Introduction

Upper respiratory tract infections are the most prevalent medical condition affecting all populations, including various age groups [1,2,3,4,5,6]. Due to the relatively mild nature of these infections and the availability of more effective modern treatments against many of them, their frequency has gradually become commonly accepted as “normal”. However, the outbreak of the COVID-19 pandemic, infections relating to which typically begin as an upper respiratory tract infection (URTI), has reinstated the old interest in infectious diseases and brought some new views on even the most common and mild URTIs [7]. In particular, the issue of preventing them has become a popular subject, as treatment of already developed infections, particularly of the viral origin, still remains relatively challenging [8,9].

Among various approaches to enhancing resistance to URTIs, maintaining good status of the upper respiratory tract mucous membranes is one of the key ones [10,11]. This approach consists of avoiding the exposure of upper respiratory tracts to irritating factors, as well as extreme coolness and dryness. Another set of factors important in URTIs prevention includes maintaining life-hygiene standards, in particular good diets, adequate resting and sleep patterns, and fresh-air physical activity [5]. Additionally, the variety of dietary supplement products have presented some degree of effectiveness in reducing the risk of URTIs. Most of them contain a variety of vitamins (especially vit. C and D), microelements, plant products, and lipids [9,12,13]. 

One of the most complex natural supplements is colostrum, commonly present on the market as a derivate of bovine colostrum (BC), a compound product with a relatively high degree of similarity to human colostrum, both in composition and influence on human organism [14,15,16,17]. Traditionally, BC was regarded to enhance the appropriate function of the immune system, which has recently been confirmed by several studies [18,19,20,21]. Most of them were clinical trials on the effectiveness of bovine colostrum in reducing the frequency of upper respiratory tract infections and gastrointestinal tract infections [22,23,24,25]. 

Although available studies generally confirm the effectiveness of bovine colostrum in preventing the URTIs occurrence, their character makes the conclusive and broad interpretation of the results difficult [20,25,26]. This is partly because the majority of these studies only included athletes; therefore, these results supposedly could not be directly applied to the regular, non-athlete population [21,22,27,28,29]. Moreover, the reports from most of these trials did not provide clear information on whether they were performed in standardized conditions, especially in regard to factors potentially influencing the URTIs occurrence [26]. These may include various factors modulating the susceptibility to infections, but also the degree of exposure to pathogens [4,5]. These exogenous factors may also strongly influence the development of URTIs in individual subjects within the tested population, who, atop of such influence, differ from each other in regard to the most important endogenous factor: efficiency of individual immunity. Therefore, in order to produce credible results, based on which conclusions applicable to possibly broadest population can be drawn, the experiments concerning the preventive medicine measures ought to be highly standardized in terms of trial conditions and population characteristics.

Considering the above issues, we performed a double-blind clinical trial assessing the effect of supplementing bovine colostrum versus placebo on the development of URTIs. We tested large size populations exposed to supposedly coherent external conditions; moreover, all the subjects participated in the trial within exactly the same timeframe. Our populations of choice have become two groups of students taking various courses at Pomeranian Medical University in Szczecin, Poland. These groups were medical students (MED) and Health Science students (HSci)—both groups differed in exposure to contact with pathogens and the workload at school. Our trial is the first ever attempt to test the relationship between colostrum supplementation and URTI occurrence in the relatively uniform adult population of a non-athletic background.

## 2. Materials and Methods

### 2.1. Recruitment, Blinding and Randomization

Trial subjects consisted of full-time students attending courses at Pomeranian Medical University in Szczecin, Poland. The only exclusion criterium was having an allergy to cow’s milk. The general health status of all participants was considered good and made them eligible for studying full time at the university, which was confirmed by obligatory periodic medical check-ups.

The trial was administered by a dedicated person, an administrator, who controlled the enrolment process and collection of formal consent forms, performed blinding and randomization process of participants, and finally, maintained the database. In the present study participants, recruiters and the study manager were blinded to allocation. The administrator was also the only person to contact participants by e-mail and to provide them with links to all the on-line survey forms (initial as well as daily/weekly surveys). These questionnaires were provided to participants based on the EUSurvey system, a free of charge, official online survey management tool launched in 2013 by the European Commission [30].

The recruitment of each participant was preceded by the detailed explanation of study principals in verbal as well as written form. Upon acquiring the formal consent forms, 271 students were recruited to the study. Of those originally enrolled, 9 participants dropped out due to suspecting being allergic to milk, 10 resigned without stating the reason and an additional 18 failed to respond to any or all of the entry level on-line surveys. Finally, 234 participants qualified to enter the study in the fall of 2021. They were blinded by associating them with individual trial ID numbers, which from this point were the only attribute used to identify the participants. Subsequently, they were stratified by attendance to certain study groups and randomly assigned to supplementation groups (with the use of MS Excel Random Sort tool). Finally, all participants began supplementation upon the signal given by the administrator. During the first three weeks of the trial, an additional 7 participants informed us about their resignation from further participation stating various, usually personal, reasons. Just two participants, both from placebo group, decided on resignation due to observing supposed side effects of the supplementation—bloating and headache.

### 2.2. Final Selection for Analysis

The unannounced drop-out of participants without stating any reason for it occurred throughout the trial period and was preceded in most cases by erratic or delayed responses to daily on-line surveys. Therefore, we decided to perform the eligibility assessment based on participation in daily surveys. In addition, upon collecting all the data, it appeared that some participants reported extremely high overall symptom severity scores of URTIs. Consequently, we assessed the validity for final analysis based on individual participants’ daily reporting performance and URTIs scores staying within acceptable limits.

In this final selection process, we decided to set a minimal limit of 85 responses to a total of 107 published daily surveys (79.4%) to prove the eligibility for final analysis. Another parameter we used was a total URTIs severity score (for definition, see below in the surveys section) of 59, which we decided to be the highest acceptable. The score of 60 indicated that participant reported at least mild symptoms of URTIs for 56% of the trial duration (60 days out of 107), or more severe symptoms for a relatively shorter amount of time. In our judgement, such a high URTI score value reflected either reckless reporting on the health status by the particular participant or the fact that such individual’s health status did not fit into description of a young, generally healthy individual.

Based on responding to at least 85 surveys, we have qualified 179 participants, of whom we additionally excluded 21 subjects due to their URTI score being higher than 59. The final number of subjects qualified for analysis was 158.

### 2.3. Study Group Characteristics

After the final selection was completed, out of the remaining 158 eligible participants, 77 were placed in the colostrum (COL) group and 81 in the placebo (PBO) group. They were enrolled in various university programs: medicine (MED) program (n = 67, 42.4%, COL—n = 32, PBO—n = 35), and 5 different Health Science (HSci) bachelor programs: nursing (n = 28, 17.7%), dietetics (n = 28, 17.72%), cosmetology (n = 13, 8.22%), physiotherapy (n = 11, 6.96%) and health psychology (n = 11, 6.96%). We analyzed all HSci groups participants together (n = 91, 57.6%, COL—n = 45, PBO—n = 46). The majority of participants (n = 123, 77.84%) were female and the median age was 21 years for both sexes. There was a difference in median age between the medical (MED) and Health Science (HSci) students (22 and 20, respectively, *p* < 0.0001).

### 2.4. Medical vs. Health Science Group

Although all participants within COL and PBO groups were subjects of identical treatment in accordance with trial protocol, we have dissected our trial groups based on the course of the study for final analysis. Thus, we obtained two major groups—MED and Hsci—which differed in some important respects, such as study workload and exposure to the infectious factors. Both of these factors were related to the course of the study in our experimental population. The workload is seemingly much larger in the medicine program than other HSci studies, which is a common opinion, fully substantiated by the experiences of our research team members—mostly medical university teachers. All of the participants in the MED group of our trial were 3rd year students and attended their practical classes in hospital units and outpatient clinics on a regular basis, which created a high risk of contact with infectious factors. The majority of our HSci students were 1st year students and had most of their classes held in lecture halls, dedicated classrooms or an online setting (due to the COVID-19 risk). According to these faculty groups’ characteristics, we obtained a group of potentially increased risk of URTIs (MED) and the control group with regular risk (HSci).

### 2.5. Supplementation Material

The supplementation packages of 90 unlabeled sachets containing either colostrum or placebo were prepared by Genactiv Trade Sp. z o. o., Poznań, Poland. Colostrum pouches contained 500 mg of a freeze-dried bovine colostrum collected within first 2 h after calf delivery was mixed with 500 mg of desiccated banana. Placebo doses were 500 mg of a spray-dried whey mixed with 500 mg of identically dried-out banana. Such an assortment of supplementation materials was effectively used in our previous experiments [31,32].

### 2.6. Design of the Trial

**Figure 1:**
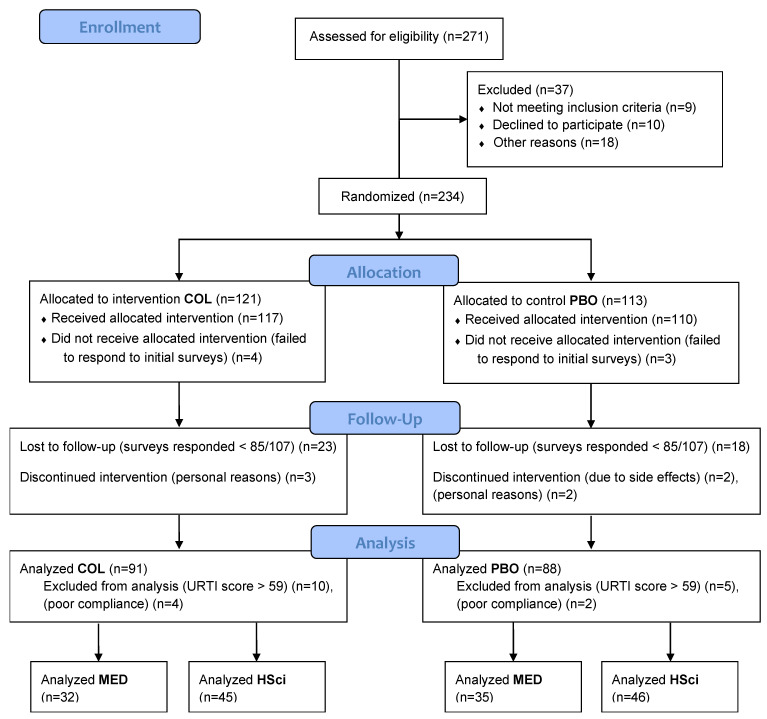
The CONSORT flow diagram.

The outline of the trial is presented on the flow chart (Figure 1). Upon completion of recruitment, blinding, and randomization, the participants received supplementation packages, individually marked with their previously assigned ID numbers. All the communication with participants, including instructions, surveys publication, and meetings with the research team members was conducted over e-mail and MS Teams. Prior to supplementation, the participants were encouraged to participate in instructional online meetings with the main researcher. They also had opportunity to watch the recorded versions of these meetings. Additionally, in this pre-supplementation period, they were asked to fill-out the initial surveys containing questions covering issues regarding their general health status and brief medical history.

The supplementation of all participants started on 7 November upon signal given by the administrator. Over the first 15 days, the participants were asked to take 2 of the 500 mg sachets of supplementation material per day. One sachet in the morning and one in the evening, taken on empty stomach with water or with a teaspoonful of natural yoghurt. Then, for the next 30 days, they were asked to use just one sachet in the morning. After 45 days, the supplementation was suspended. The participants were asked to take 4 consecutive doses of supplementation material (twice daily for 2 days) just in case they observed symptoms of developing a URTI. Supplementation with 2 sachets per day was reinstated for an additional 7 days one week before the semester ended (at the day 87 of the trial), when most of the tests and exams concluding the semester would take place. After this last supplementation period, the daily reports were collected for an additional 14 days, and on 21 February, 107 days from beginning of supplementation, the trial was terminated.

### 2.7. The Surveys

All the information gathered on the health status and behavior of trial participants was collected based on the online surveys. The surveys were delivered to participants with the EUSurvey tool (https://ec.europa.eu) through direct links sent via e-mail with the results collected individually from each participant. Every survey questionnaire was kept available for responding or editing over 7 days from publication date.

There were three types of surveys used in this trial: initial, daily, and weekly. The initial trial was devoted to obtaining data used for descriptive statistics, as well as the information helping to develop the overview characteristic of the tested population. Questions on medical history and dietary, as well as lifestyle, habits regarding period from delivery up to present time were included in this survey.

The most important aspect for gathering data analyzed in our experiment was daily surveys. The daily survey questionnaire was brief (7 questions) and the main questions concerning the health status of participants were those about the severity of URTI symptoms, presence, and type of gastrointestinal (GI) symptoms, as well as well-being status. 

The question about the presence and severity of URTIs symptoms offered a choice of four responses with the following scores: 0 points—no symptoms; 1 point—mild symptoms not influencing regular daily activity (sporadic sneezing and/or cough, mild sore throat, runny/blocked nose of low intensity—at least one of these symptoms with no fever); 2 points—mediocre symptoms reducing regular daily activity (cough, runny/blocked nose, pain in area of sinuses—at least one of these symptoms with body temperature < 38.0 °C); 3 points—severe symptoms incapacitating regular daily activity (Ist group of symptoms: persisting cough, runny/blocked nose of significant intensity, sinus ache, IInd group of symptoms: serious headache, muscle ache, skin sensitivity to touch, loss of appetite, thermic discomfort, fever of ≥38.0 °C—combination of at least one from the Ist group and two from the IInd group of symptoms).

Additionally, also from daily surveys, the question regarding well-being contained a choice of four responses scored as follows: 3 points—perfect (cannot be any better); 2 points—good (all is okay, no complaints); 1 point—mediocre (feel passably, but could be better); 0 points—poor (something is not right, in a bad mood).

This way, the numerical scales were created which allowed us to grade the intensity of such subjective parameters as severity of self-observed symptoms and well-being. The values obtained from each participant were summed up to create scores on these variables, analyzed as the overall result from the entire study as well as data broken down to various periods of the trial, i.e., days 1–45 (the initial supplementation period), 46–86 (no supplementation), 87–107 (second supplementation and 2 weeks follow-up).

The most important question in our weekly surveys was about the number of days with any URTIs symptoms observed over the last seven days.

### 2.8. Statistical Analysis

The distribution of continuous variable was verified with Shapiro–Wilk test. As the majority of variables were significantly different form normal distribution, they were presented as medians (M) and interquartile ranges (IQR). Qualitative data were expressed as numbers and percentages. To test whether the allocation procedure resulted in differences in gathered data, Mann–Whitney/Kruskal–Wallis or chi2/Fisher’s exact test were used when appropriate. To evaluate the interaction between tested groups allocation and faculty, two-way ANOVA was used; however, data were ranked, if necessary, to conform to normality. The level of significance was set as *p* < 0.05, while *p* = 0.05–0.1 was considered as the area of the statistical trend. Statistical analysis was performed using the MedCalc software ver. 20.110 (Ostend, Belgium).

### 2.9. Bioethical Approval

The trial was performed in accordance with the protocol approved by the Pomeranian Medical University Bioethics Committee (KB-0012/25/21) on 28 June 2021.

## 3. Results

### 3.1. Participants Data

Trial participants within particular studied groups were well balanced in terms of medical history issues which may have influenced immunity status. The majority of trial participants were born by vaginal delivery (COL: n = 52, 67.5%; PBO: n = 44, 54.3%; *p* = 0.22) and breast fed (COL: n = 59, 76.6%; PBO: n = 55, 67.9%; *p* = 0.36). There were no significant differences regarding the ailments and pharmacotherapy in the childhood/adolescence periods, as well as during the three years preceding the trial enrolment between the COL and PBO groups (Table 1). There were 7 persons in each group with COVID-19 confirmed by PCR test during trial (*p* = 0.92). Most of the study participants had received COVID-19 vaccine (COL: n = 74, 96.1% and PBO: n = 75, 92.6%; *p* = 0.34). Equal number of participants (n = 25) in both COL (32.5%) and PBO (30.9%) groups were vaccinated against influenza at least once in a lifetime.

### 3.2. Colostrum Efficacy in Mitigating Frequency of URTIs

**Figure 2:**
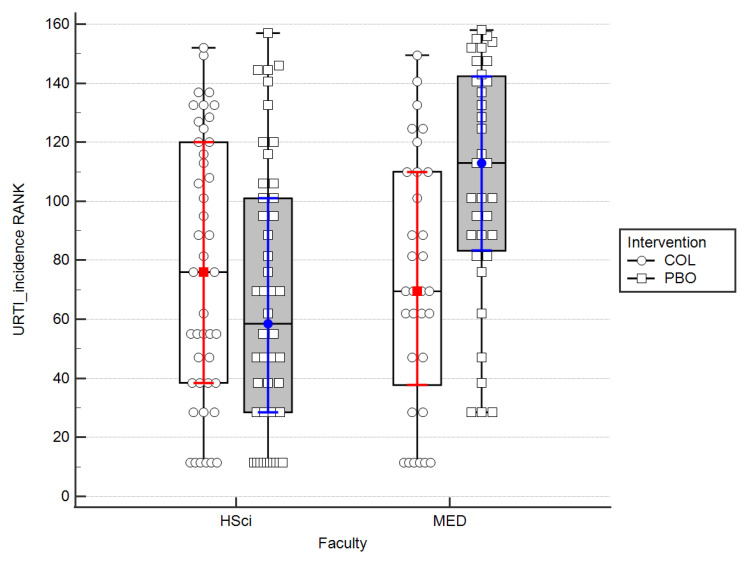
A bar plot on the efficacy of COL in reducing of the URTIs symptoms frequency by the course of studies. Medians and IQRs are shown. Dots and squares depict individual cases; data are ranked. MED—medicine; HSci—health Science.

To assess the efficacy of colostrum (COL) in reducing the frequency of URTIs, we analyzed the number of days in which participants observed any symptoms of URTIs based on weekly reports over the entire experiment period (107 days). Analyzing all participants together, we found an insignificantly lower number of days with any URTIs symptoms in subjects receiving colostrum compared to those receiving placebo (COL: 6 (2–15.25) vs. PBO: 9 (2–18.5); *p* = 0.21). However, when we analyzed the influence of supplement vs. placebo in regard to faculty (MED vs. HSci), it turned out that the intervention with colostrum significantly diminished the number of days with symptoms of URTIs in the MED group. We found a tendency towards statistically significant difference by intervention (f(1) = 3.006, *p* = 0.085), a significant difference by faculty (f(1) = 6.53, *p* = 0.012), and a significant interaction between these variables (f(1) = 10.42, *p* = 0.002; Figure 2).

**Figure 3:**
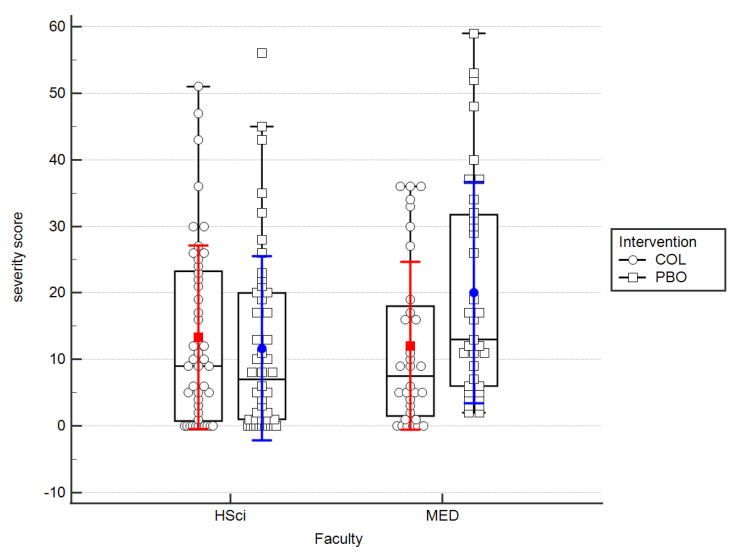
A bar plot on the efficacy of COL in reducing of the URTIs severity by the course of studies. Medians and IQRs are shown. Dots and squares depict individual cases. MED—medicine; HSci—health Science.

When we summed up the URTIs symptoms severity results and analyzed them collectively in all trial participants within both intervention groups (COL and PBO) over the entire observation period, these scores were higher in the placebo group, but insignificantly (COL: 9 (1–22.5) vs. PBO: 11 (2.75–23.75; *p* = 0.31). When we split the participants according to faculty (medicine vs. others) though, we were able to demonstrate insignificant interaction between the independent variables (by intervention (f(1) = 1.91, *p* = 0.169), by faculty (f(1) = 2.37, *p* = 0.126) and a statistically significant interaction between these variables (f(1) = 4.51, *p* = 0.035; Figure 3).

The frequency of days with any URTIs symptoms, as reported weekly, and severity scores obtained from daily surveys were also analyzed by two-way analyses of variance. Data divided into periods of the trial (supplementation—days 1–45, no supplementation—days 46–86 and secondary supplementation plus observation in the final two weeks—days 87–107) are presented in Table 2.

### 3.3. Colostrum Efficacy in Improving Daily Well-Being Perception

**Figure 4:**
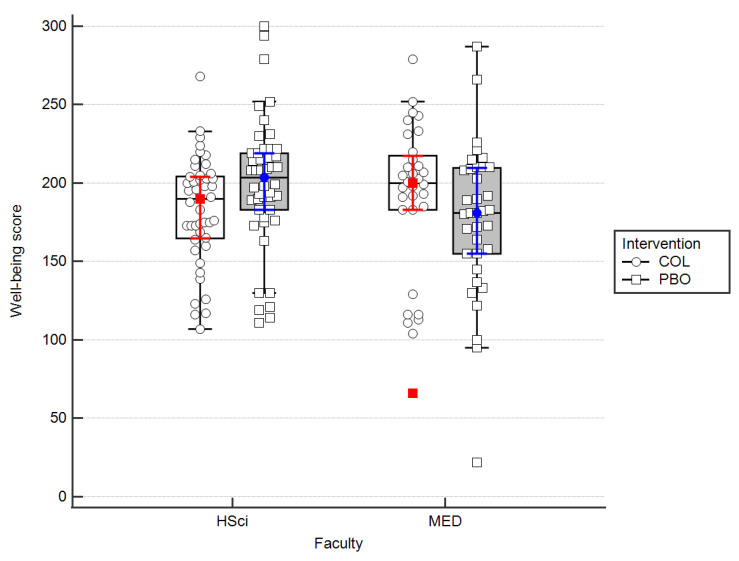
A bar plot on the efficacy of COL in shaping well-being score by the course of studies. Medians and IQRs are shown. Dots and squares depict individual cases. MED—medicine; HSci—health Science.

The comparison of well-being scores from daily reports has demonstrated no difference between the tested groups when all the participants were analyzed collectively (COL: 196 (168–210.25) vs. PBO: 192 (171.75–215.25); *p* = 0.61). However, similarly to URTIs severity, significant interactions between independent variables were demonstrated when analyzed over entire study length as well as in its particular periods (Table 3, Figure 4).

### 3.4. Adverse Events

There were no serious adverse events reported during the entire trial. The GI symptoms during first 45 days (the first and the longest supplementation period) and the whole study time did not differ significantly, either by allocation or by faculty, as shown in Table 4.

## 4. Discussion

The popularity of bovine colostrum as a health supplement has substantially grown in the recent years, urging the need for more credible proofs of its effectiveness. Various aspects of its supposed advantageous influence on human health have been tested in numerous laboratory and clinical experiments, the results of which mostly confirmed these claims [15,16,17,33]. One very important benefit most typically attributed to colostrum has always been the improvement of immunity, which is best expressed as the reduction in respiratory tract infection frequency and/or severity. This was tested in several experiments, some of which have been double-blind placebo-controlled (DBPC) trials, performed mostly with athletes or at least physically active participants [18,21,27,28,34]. Although their results were mostly in favor of colostrum preventing the respiratory tract infectious events, the choice of trial subjects and other parameters have made their results difficult to apply to the general, non-athlete population.

Our results come from the first ever DBPC trial with such large, highly uniform adult non-athletic population, performed in one location, and within the same timeframe to produce most coherent epidemiological conditions. All previous trials testing the influence of colostrum the on development of URTIs differed from our work in regard to some of these experimental design characteristics [18,21,27,28,34,35]. We made this effort because, as much as keeping such strict experimental conditions is challenging, it is crucial for obtaining credible and accurate results, especially with epidemiological trials assessing prevention from infectious diseases.

An additional factor that differs our study from previous trials is that we used much lower doses of colostrum. Most of the other experiments of similar design used daily doses of 10–60 g per day, whereas our doses were 500 mg twice daily followed by 500 mg once daily. Such low doses were previously tested in our two trials and were aimed at downregulating intestinal permeability in athletes and they turned out to be highly effective in this regard [31,32].

An additional exclusive feature of our study is that the observation period was longer than in other trials and lasted for more than 15 weeks (107 days). In other trials of the same type the participants were monitored for 8 to 14 weeks [18,21,27,28,34]. Moreover, supplementation in our trial was applied in two separate courses lasting for 45 and 7 days, whereas in the majority of other experiments, the supplementation lasted over the entire observation period. Only in two trials the observation was extended for two weeks beyond the supplementation time [27,28]. In our trial we had two periods of observation without simultaneous supplementation—one of 41 days in the middle of the active trial period and another of 2 weeks by the end of it.

### 4.1. The URTIs Frequency and Severity

The most important parameters tested in our trial were frequency and severity of URTIs. These parameters are considered to best reflect the effectiveness of the immune system and belong to the group of major indicators of immunodeficiency in clinical practice. However, they depend not only on the individual’s immunity status. The frequency of URTIs is strongly related to intensity of exposure to infectious agents, which may be affected by the seasonal epidemiological changes and the nature of the individual’s social interactions with the environment. On the other hand, the frequency, as well as the severity of URTIs, also depend importantly on the general physical fitness, which may be affected by heavy workload exhausting the energetic and structural resources, which are crucial for appropriate immunity status.

Previously, the experiments with colostrum supplementation applied to usually “overworked” and exhausted trial participants, such as professional athletes, were consistently successful in improving protection from URTIs [21,22,27,28,32]. There was one experiment, however, where regular students with no athletic background were used as a control population for athletes and the results in this group showed no difference between the PLA and COL groups [28]. This suggests that benefits from colostrum use in regard of URTIs protection can be observed in those whose naturally occurring resistance to infections can be exposed to exceptional challenges, such as physical or possibly also mental strains.

In our trial we selected two groups of participants: “normal” population of healthy young people as control (health science students—HSci) and the “at risk” population of their hard-working peers (medical students—MED)—the exposure to infectious agents and the workload parameters differed in both. Studying medicine is known to be related to a much higher load of various duties and stressful situations compared to HSci studies, thus it can be regarded an extreme condition that increases the risk of URTIs in a fashion parallel to athletic activity.

In addition to the differences resulting from a variety in workload related to the course of the study, these subpopulations attended theoretical courses in separate university venues. Moreover, the academic activities of medical students were predominantly held in healthcare facilities (hospital units and outpatient clinics), whereas HSci students practically did not access such facilities for their lessons. This was mainly because all medical students were third year students who have plenty of practical classes, whereas majority of HSci courses students were first year students, participating mainly in theoretical courses. All these have caused high heterogeneity in risk of exposure to infectious agents between these two subpopulations. 

Dissecting our participants into two independent populations (HSci and MED) allowed us to maintain high homogeneity of such created groups in both workload and risk of exposure, which was confirmed by the trial results. There were numerous differences between groups in regard to URTI frequency and severity both between untreated groups (PBO) as well as in reaction to colostrum supplementation (COL) groups.

When we analyzed the entire population of students without dissecting them into HSci and MED groups over the whole trial period, the results regarding the frequency and severity of URTIs showed no statistically significant difference between the colostrum and placebo groups (*p* = 0.085). This stood in contrast with the majority of the results published by other authors, except for the trial mentioned above, where athletes were tested predominantly [18,21,27,28,34]. When we analyzed the results from PBO and COL groups within the HSci versus MED populations in two-way analyses of variance, it turned out that statistically significant effectiveness of colostrum in prevention of URTIs frequency (*p* = 0.002) (Figure 2) and severity (*p* = 0.035) (Figure 3) could be observed.

Other interesting findings appeared when we analyzed the URTIs symptoms days frequency after dissecting our experiment into three distinct periods: period I—days 1–45—supplementation, period II—days 46–86—no regular supplementation and period III—days 87–107—final one-week long supplementation and two weeks of follow up period. We found statistically significant differences between PBO and COL in MED vs. HSci group in all three periods of the study (periods I, II and III, respectively, *p* = 0.034, *p* = 0.034 and *p* = 0.014) (Table 2). However, in period III we also found that in COL, group participants were developing URTIs significantly less frequently than in the PBO group, even when the global population of students in our trial was analyzed (*p* = 0.029). This suggests that after applying the repeated course of supplementation starting by day 87 of the trial, the level of protection from URTIs increased to reach an important level in participants not being at increased risk of infection. Such effect has never been observed before, since previous studies used continuous supplementation and this may potentially indicate that interval supplementation of colostrum can increase its effectiveness. 

Parallel analysis of URTIs severity in individual periods of the trial has brought no statistically significant results in regard to the difference between COL and PBO groups; in both, global population of students was analyzed when divided into HSci and MED groups.

Our results regarding decrease in frequency and severity of URTIs symptoms upon consuming colostrum in students show clearly that healthy young people can benefit from this kind of supplementation only when they become exposed to a higher risk of developing these infections, such as with participants from the MED group in our experiment. 

There was relatively low frequency of URTIs over 3.5 fall/winter months of our trial with only 10.6 days with URTIs symptoms (including the mildest) on average, which was 1.11 URTIs episodes per person, whereas the expected frequency is between 2 to 4 episodes per year [1]. This resulted not only from high resistance to infections naturally occurring in our young participants but was also most probably the effect of a high level of anti-infectious protection among our health professionals in training, which included social distancing and face masks wearing. The trial was conducted between November 2021 and February 2022, when the epidemiological risks due to COVID-19 were still high. This was a weak point of our trial, probably preventing us from reaching the statistical significance in some regards. To obtain most convincing statistical results from epidemiological studies concerning such relatively infrequent events, much larger populations need to be included in the trial.

### 4.2. URTIs Episodes

The traditional understanding of URTI is that it appears as a streak of consecutive days with upper respiratory tract symptoms, usually lasting for 3 to 7 or more days, which can be defined as infectious episode [36,37]. Some studies report on the frequency of such episodes among their participants, usually offering their own definition of such episode, which in most cases is based on the defined number of consecutive days with symptoms and symptomless period required to separate individual episodes [18,34,35]. Usually there is little or nothing stated about how severe these symptoms have to be in order to include the particular day into episode.

Analyzing our data, we initially attempted to process the results in the way that would allow us to present the statistics on the number of episodes. However, despite collecting very precise daily reports with the level of severity stated for each upper respiratory symptoms set appearance, we were not able to clearly define the URTIs episodes, at least among substantial proportion of our participants. These streaks of symptomatic days were either too long for a typical period of non-chronic respiratory tract infection, were separated by symptomless periods that were too short (less than 3 days), or their severity score was very mild.

It seems that reporting URTIs symptoms on daily basis may have become too sensitive a parameter in regard to judging whether the self-registered symptoms were actual signs of the infection or they were just casually appearing, meaningless events (occasional sneezing, for instance). This is why we decided to assess the actual infectious days frequency based on weekly, not daily reports. Reporting weekly gives us a chance to fully assess the meaning of the symptoms and decide whether the particular day was infectious one or not.

A high degree of diversity of individual URTIs episodes appearance and their potentially unexpected course can occur for several reasons. One could be having a large variety and still changing the spectrum of infectious agents causing these infections [3,4,5,38,39]. Another is individual susceptibility to these infections, frequently modulated by various vaccinations and other immunomodulatory treatments. Finally, probably the most important factor causing the URTIs episodes to look unusual, is very frequent use of symptomatic treatment medications, mostly available over the counter. These medications may not stop the disease but can effectively interrupt or a least sooth the presence of disease symptoms. Requiring participants to refrain from the use of these medications over a prolonged period of time in such large trials is very hard to achieve.

Consequently, categorizing the symptomatic days into episodes in our opinion is extremely subjective and can produce high bias level in both reporting and analyzing data. Thus, for reliability of the statistics on URTIs, the trial results should preferably be presented as the number of symptomatic days and the severity scores, as we did in this report.

### 4.3. Well-Being Change upon Supplementation

Important data collected in our trial included the well-being reports from all of the students. We found out that, similarly to URTIs symptoms frequency and severity change upon colostrum supplementation, the well-being changes were not statistically significant between COL and PBO when all students were analyzed collectively. However, when we split them according to faculty, the statistically significant improvement of well-being upon supplementation with colostrum was found in MED vs. HSci groups. This was confirmed by two-way ANOVA for entire length of experiment (*p* = 0.037) (Figure 4), as well as being found nearly significant in period I (*p* = 0.056) and significant in period III (*p* = 0.01) (Table 3). According to our best knowledge, our data on well-being improvement upon colostrum supplementation are the first published to date report of this kind.

### 4.4. Side Effects Monitoring

Another valuable type of information from our trial, which stands in accordance with what has already been reported, is data on possible adverse effects [40]. In the past it has been observed that the only possible negative side effects upon oral colostrum use were symptoms from gastrointestinal tract, with nausea diarrhea, bloating, and abdominal pain [17,40]. These are suggested to develop usually at the beginning of supplementation and disappear after few days. None of the above side effects have appeared in significantly larger frequency in COL group than in PBO group in our trial (Table 4). There were no important adverse effects of any other type reported during the entire trial as well.

## 5. Conclusions

Our trial brings new insight to the issue of colostrum effectiveness by providing evidence that in contrast to general healthy population of young adults, subpopulation of those exposed to certain challenges which increase the risk of developing URTIs, can significantly benefit from using colostrum supplementation. To confirm this general notion, several particular conclusions can be drawn from our trial:

1. Colostrum supplementation significantly decreases number of days with symptoms of URTIs registered in young healthy population at increased risk of developing URTIs (MED group) versus population of their peers with no elevated risk of such infections (HSci group).
2. The reduction in severity of URTIs symptoms is observed upon supplementation with colostrum in MED versus HSci group as well.
3. Well-being is also significantly improved in the MED group supplemented with colostrum when compared with such supplemented HSci group.
4. The above effects can be obtained with much smaller doses than in previous trials of parallel design.
5. There were no serious side effects among those receiving colostrum in all participants of our trial, as well as none of the gastrointestinal symptoms have appeared among them with higher frequency than in PBO group.

## Figures and Tables

**Table 1:** The frequency of childhood diseases in study participants by allocation procedure.

URTIs in Childhood/Youth Per Year									
	0–1	2–3	4–5	>5	*p*				
COL	23	38	14	2	0.21				
PBO	26	40	8	7					
**Surgery in childhood/youth**									
	**Abdominal/cancer**	**Abdominal**	**Orthopedic**	**Orthopedic/abdominal**	**None**	***p***			
COL	0	3	10	1	63	0.49			
PBO	1	4	6	0	70				
**Serious ailments in childhood/youth**									
	**Autoimmune**	**Cancer**	**Infections**	**Infections/autoimmune**	**Infections/autoimmune/injury**	**Infections/injury**	**Injury**	**None**	***p***
COL	1	0	5	1	0	2	0	68	0.21
PBO	4	1	1	1	1	0	1	72	
**Chronic Disease at present**									
	**Allergy**	**Allergy/autoimmune**	**Autoimmune**	**Autoimmune/diabetes**	**Autoimmune/IBD**	**Diabetes**	**IBD**	**None**	***p***
COL	2	0	0	0	0	0	2	72	0.52
PBO	2	1	2	1	1	1	1	72	
**Mean number of ABX course in the last 3 autumn winter seasons**									
	**1**	**2**	**3**	**4**	***p***				
COL	63	12	1	1	0.94				
PBO	67	11	2	1					
**Mean number of URTI in the last 3 autumn winter seasons**									
	**1**	**2**	**3**	**4**	***p***				
COL	31	37	7	2	0.61				
PBO	27	40	9	5					
**Prolonged (>2 weeks) ABX during last 3 months**									
	**NO**	**YES**	***p***						
COL	74	3	0.95						
PBO	78	3							

COL—colostrum, PBO—placebo, IBD—inflammatory bowel disease, ABX—antibiotic.

**Table 2:** The interaction between group allocation and faculty for URTIs symptoms days and severity as reported daily over three periods of the study. For two-way analysis data on URTIs incidence are ranked.

Variable	Colostrum			Placebo			*p*	Factor ^R^	DF	F	*p*
	n	Median	IQR	n	Median	IQR					
URTIs number of days 1–45	77	2	0.000–6.000	81	3	1.000–9.000	0.1594	Intervention	1	2.953	0.088
								Faculty	1	2.285	0.133
								Intervention*Faculty	1	4.582	0.034
URTIs number of days 46–86		1	0.000–5.000		1	0.000–6.250	0.8113	Intervention	1	0.3	0.585
								Faculty	1	0.922	0.338
								Intervention*Faculty	1	4.576	0.034
URTIs number of days 87–107		0	0.000–1.250		0	0.000–3.250	0.069	Intervention ^R^	1	4.887	0.029
								Faculty ^R^	1	6.472	0.012
								Intervention ^R^ *Faculty	1	6.197	0.014
URTIs severity score days 1–45		4	0.000–9.000		4	0.000–12.250	0.3604	Intervention	1	1.353	0.247
								Faculty	1	3	0.085
								Intervention*Faculty	1	3.275	0.072
URTIs severity score days 46–86		0	0.000–6.250		1	0.000–6.500	0.8459	Intervention	1	0.106	0.746
								Faculty	1	0.617	0.433
								Intervention*Faculty	1	0.929	0.337
URTIs severity score days 87–107		0	0.000–1.000		0	0.000–2.000	0.5264	Intervention	1	0.691	0.407
								Faculty	1	4.656	0.033
								Intervention*Faculty	1	2.316	0.13

^R^—data are ranked, DF—degrees of freedom.

**Table 3:** The interaction between allocation and faculty for well-being score in three periods as well as over the entire study.

Variable	Colostrum			Placebo			*p*	Factor	DF	F	*p*
	n	Median	IQR	n	Median	IQR					
Well-being score	77	196	168.000–210.250	81	192	171.750–215.250	0.6127	Intervention	1	0.0578	0.81
								Faculty	1	1.327	0.251
								Intervention*Faculty	1	4.414	0.037
Well-being score days 1–45		81	71.500–88.000		82	71.000–90.000	0.4164	Intervention	1	0.012	0.913
								Faculty	1	0.608	0.437
								Intervention*Faculty	1	3.721	0.056
Well-being score days 46–86		75	62.000–81.000		76	61.500–83.000	0.7941	Intervention	1	0.0272	0.869
								Faculty	1	1.775	0.185
								Intervention*Faculty	1	2.108	0.149
Well-being score days 87–107		39	30.500–42.000		39	31.750–43.000	0.4529	Intervention	1	0.235	0.628
								Faculty	1	0.877	0.351
								Intervention*Faculty	1	6.771	0.01

DF—degrees of freedom.

**Table 4:** GI symptoms per person during first 45 days or whole trial (WT) period by faculty and allocation.

Variable	Colostrum			Placebo			*p*
	n	Median	IQR	n	Median	IQR	
Bloating_45days	77	0	0.000–2.000	81	1	0.000–2.250	0.7104
Bloating_WT		1	0.000–2.000		1	0.000–4.000	0.7437
Diarrhea_45days		0	0.000–2.000		0	0.000–1.000	0.2605
Diarrhea_WT		1	0.000–3.000		0	0.000–2.000	0.2437
Pain_45days		1	0.000–4.000		1	0.000–5.000	0.6648
Pain_WT		2	0.000–6.000		2	0.000–7.250	0.7909
No symptoms_45days		41	37.000–44.000		40	35.000–44.000	0.9624
No symptoms_WT		88	81.750–93.000		89	80.750–93.000	0.8836
MED				HSci			
Bloating_45days	67	1	0.000–2.000	91	0	0.000–2.000	0.3787
Bloating_WT		1	0.000–3.000		0	0.000–3.000	0.2871
Diarrhea_45days		0	0.000–2.000		0	0.000–2.000	0.7103
Diarrhea_WT		1	0.000–2.750		0	0.000–2.750	0.3133
Pain_45days		1	0.000–4.000		2	0.000–4.000	0.1596
Pain_WT		2	0.000–5.000		3	0.000–7.000	0.2452
No symptoms_45days		41	37.250–44.000		40	35.000–44.000	0.3835
No symptoms_WT		89	83.000–93.000		88	78.500–92.750	0.5019

WT—whole trial.

## Data Availability

Upon request.
